# Transient Cognitive Dynamics, Metastability, and Decision Making

**DOI:** 10.1371/journal.pcbi.1000072

**Published:** 2008-05-02

**Authors:** Mikhail I. Rabinovich, Ramón Huerta, Pablo Varona, Valentin S. Afraimovich

**Affiliations:** 1 Institute for Nonlinear Science, University of California San Diego, La Jolla, California, United States of America; 2 Grupo de Neurocomputación Biológica, Department of Ingeniería Informática, Universidad Autónoma de Madrid, Madrid, Spain; 3 Instituto de Investigación en Comunicación Óptica, UASLP, San Luis de Potosí, Mexico; University College London, United Kingdom

## Abstract

The idea that cognitive activity can be understood using nonlinear dynamics has been intensively discussed at length for the last 15 years. One of the popular points of view is that metastable states play a key role in the execution of cognitive functions. Experimental and modeling studies suggest that most of these functions are the result of transient activity of large-scale brain networks in the presence of noise. Such transients may consist of a sequential switching between different metastable cognitive states. The main problem faced when using dynamical theory to describe transient cognitive processes is the fundamental contradiction between reproducibility and flexibility of transient behavior. In this paper, we propose a theoretical description of transient cognitive dynamics based on the interaction of functionally dependent metastable cognitive states. The mathematical image of such transient activity is a stable heteroclinic channel, i.e., a set of trajectories in the vicinity of a heteroclinic skeleton that consists of saddles and unstable separatrices that connect their surroundings. We suggest a basic mathematical model, a strongly dissipative dynamical system, and formulate the conditions for the robustness and reproducibility of cognitive transients that satisfy the competing requirements for stability and flexibility. Based on this approach, we describe here an effective solution for the problem of sequential decision making, represented as a fixed time game: a player takes sequential actions in a changing noisy environment so as to maximize a cumulative reward. As we predict and verify in computer simulations, noise plays an important role in optimizing the gain.

## Author Summary

The modeling of the temporal structure of cognitive processes is a key step for understanding cognition. Cognitive functions such as sequential learning, short-term memory, and decision making in a changing environment cannot be understood using only the traditional view based on classical concepts of nonlinear dynamics, which describe static or rhythmic brain activity. The execution of many cognitive functions is a transient dynamical process. Any dynamical mechanism underlying cognitive processes has to be reproducible from experiment to experiment in similar environmental conditions and, at the same time, it has to be sensitive to changing internal and external information. We propose here a new dynamical object that can represent robust and reproducible transient brain dynamics. We also propose a new class of models for the analysis of transient dynamics that can be applied for sequential decision making.

## Introduction

The dynamical approach for studying brain activity has a long history and is currently one of strong interest [1]–[7]. Cognitive functions are manifested through the generation and transformation of cooperative modes of activity. Different brain regions participate in these processes in distinct ways depending on the specific cognitive function and can prevail in different cognitive modes. Nevertheless, the mechanisms underlying different cognitive processes may rely on the same dynamical principles, e.g., see [8].

The execution of cognitive functions is based on fundamental asymmetries of time – often metaphorically described as the arrow of time. This is inseparably connected to the temporal ordering of cause-effect pairs. The correspondence between causal relations and temporal directions requires specific features in the organization of cognitive system interactions, and on the microscopic level, specific network interconnections. A key requirement for this organization is the presence of nonsymmetrical interactions because, even in brain resting states, the interaction between different subsystems of cognitive modes also produces nonstationary activity that has to be reproducible. One plausible mechanism of mode interaction that supports temporal order is nonreciprocal competition. Competition in the brain is a widespread phenomenon (see [9] for a remarkable example in human memory systems). At all levels of network complexity, the physiological mechanisms of competition are mainly implemented through inhibitory connections. Symmetric reciprocal inhibition leads to multistability and this is not an appropriate dynamical regime for the description of reproducible transients. As we have shown in [5],[10], nonsymmetric inhibition is an origin of reproducible transients in neural networks.

Recently functional magnetic-resonance imaging (fMRI) and EEG have opened new possibilities for understanding and modeling cognition [11]–[15]. Experimental recordings have revealed detailed (spatial and temporal) pictures of brain dynamics corresponding to the temporal performance of a wide array of mental and behavioral tasks, which usually are transient and sequential [16]–[18]. Several groups have formulated large-scale dynamical models of cognition. Based on experimental data these models demonstrate features of cognitive dynamics such as metastability and fast transients between different cognitive modes [15], [16], [19]–[24]. There is experimental evidence to support that metastability and transient dynamics are key phenomena that can contribute to the modeling of cortex processes and thus yield a better understanding of a dynamical brain [18], [25]–[30].

Common features of many cognitive processes are: (i) incoming sensory information is coded both in space and time coordinates, (ii) cognitive modes sensitively depend on the stimulus and the executed function, (iii) in the same environment cognitive behavior is deterministic and highly reproducible, and (iv) cognitive modes are robust against noise. These observations suggest (a) that a dynamical model which possesses these characteristics should be strongly dissipative so that its orbits rapidly “forget” the initial state of the cognitive network when the stimulus is present, and (b) that the dynamical system executes cognitive functions through transient trajectories, rather than attractors following the arrow of time. In this paper we suggest a mathematical theory of transient cognitive activity that considers metastable states as the basic elements.

This paper is organized as follows. In the Results section we first provide a framework for the formal description of metastable states and their transients. We introduce a mathematical image of robust and reproducible transient cognition, and present a basic dynamical model for the analysis of such transient behavior. Then, we generalize this model taking into account uncertainty and use it for the analysis of decision making. In the Discussion, we focus on some open questions and possible applications of our theory to different cognitive problems. In the Methods section, a rigorous mathematical approach is used to formulate the conditions for robustness and reproducibility.

## Results

### Metastability and Cognitive Transient Dynamics

**Figure 1:**
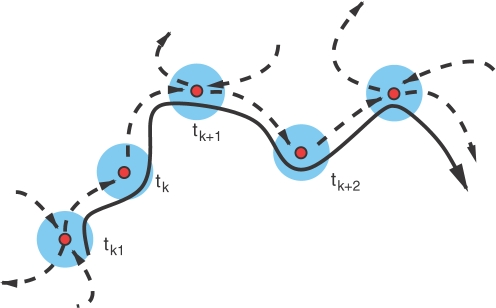
Schematic representation of a stable heteroclinic channel. The SHC is built with trajectories that condense in the vicinity of the saddle chain and their unstable separatrices (dashed lines) connecting the surrounding saddles (circles). The thick line represents an example of a trajectory in the SHC. The interval *t_k_*_+1_−*t_k_* is the characteristic time that the system needs to move from the metastable state *k* to the *k*+1.

A dynamical model of cognitive processes can use as variables the activation level *A_i_*(*t*)≥0 of cognitive states (*i* = 1…*N*) of specific cognitive functions [31]. The phase space of such model is then the set of *A_i_*(*t*) with a well-defined metric where the trajectories are sets of cognitive states ordered in time. To build this model, we introduce here several theoretical ideas that associate metastable states and robust and reproducible transients with new concepts of nonlinear dynamics, i.e., stable heteroclinic sequences and heteroclinic channels [4], [5], [10], [32]–[34]. The main ideas are the following:

- &SetFont Typeface="12";Metastable states of brain activity can be represented in a high-dimensional phase space of a dynamical model (that depends on the cognitive function) by saddle sets, i.e., saddle fixed points or saddle limit cycles.
- &SetFont Typeface="12";In turn, reproducible transients can be represented by a stable heteroclinic channel (SHC), which is a set of trajectories in the vicinity of a heteroclinic skeleton that consists of saddles and unstable separatrices that connect their surroundings (see Figure 1). The condensation of the trajectories in the SHC and the stability of such channel are guaranteed by the sequential tightness along the chain of the saddles around a multi-dimensional stable manifold. The SHC is structurally stable in a wide region of the control parameter space (see Methods).
- The SHC concept is able to solve the fundamental contradiction between robustness against noise and sensitivity to the informational input. Even close informational inputs induce the generation of different modes in the brain. Thus, the topology of the corresponding stable heteroclinic channels sensitively depends on the stimuli, but the heteroclinic channel itself, as an object in the phase space (similar to traditional attractors), is structurally stable and robust against noise.

Based on these ideas we model the temporal evolution of alternating cognitive states by equations of competitive metastable modes. The structure of these modes can be reflected in functional neuroimage experiments. Experimental evidence suggests that for the execution of specific cognitive functions the mind recruits the activity from different brain regions [35]–[37]. The dynamics of such networks is represented by sequences of switchings between cognitive modes, i.e., as we hypothesize, a specific SHC for the cognitive function of interest.

### Mathematical Image and Models

We suggest here that the mathematical image of reproducible cognitive activity is a stable heteroclinic channel including metastable states that are represented in the phase space of the corresponding dynamical model by saddle sets connected via unstable separatrices (see Figure 1). Note that the topology of Figure 1 reminds a ‘chaotic itinerancy’ [38]. However, based only on Milnor attractors we cannot demonstrate the reproducibility phenomena which is the main feature of the SHC.

To make our modeling more transparent let us use as an example the popular dynamical image of rhythmic neuronal activity, i.e., a limit cycle. At each level of complexity of a neural system, its description and analysis can be done in the framework of some basic model like a phase equation. The questions that can be answered in this framework are very diverse: synchronization in small neuronal ensembles like CPGs, generation of brain rhythms [39], etc. Our approach here is similar. We formulate a new paradigm for the mathematical description of reproducible transients that can be applied at different levels of the network complexity pyramid. This paradigm is the Stable Heteroclinic Channel. As a limit cycle, the SHC can be described by the same basic equation on different levels of the system complexity. The sense of the variables *A_i_*(*t*)≥0, of course, is different at each level.

Before we introduce the basic model for the analysis of reproducible transient cognitive dynamics, it is important to discuss two general features of the SHC that do not depend on the model. These are: (i) the origin of the structural stability of the SHC, and (ii) the long passage time in the vicinity of saddles in the presence of moderate noise.

To understand the conditions of the stability of SHC we have to take into account that an elementary phase volume in the neighborhood of a saddle is compressed along the stable separatrices and it is stretched along an unstable separatrix. Let us to order the eigenvalues of a saddle as
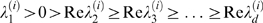
The number 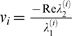 is called the saddle value. If *v_i_*>1 (the compressing is larger than the stretching), the saddle is named as a dissipative saddle. Intuitively it is clear that the trajectories do not leave the heteroclinic channel if all saddles in the heteroclinc chain are dissipative. A rigorous analysis of the structural stability of the heteroclinic channel supports our intuition (see Methods).

The problem of the temporal characteristics of the transients is related to the “exit problem” for small random perturbations of dynamical systems with saddle sets. This problem was first solved by Kifer [40] and then discussed in several papers, in particular, in [41]. A local stability analysis in the vicinity of a saddle fixed point allows us to estimate the time that the system spends in the vicinity of the saddle:
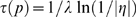
(1)where τ (*p*) is the mean passage time, |η| is the level of noise, and λ is an eigenvalue corresponding to the unstable separatrix of the saddle.

A biologically reasonable model that is able to generate stable and reproducible behavior represented in the phase space by the SHC has to (i) be convenient for the interpretation of the results and for its comparison with experimental data, (ii) be computationally feasible, (iii) have enough control parameters to address a changing environment and the interaction between different cognitive functions (e.g., learning and memory). We have argued that the dynamical system that we are looking for has to be strongly dissipative and nonlinear. For simplicity, we chose as dynamical variables the activation level of neuronal clusters that consist of correlated/synchronized neurons. The key dynamical feature of such models is the competition between different metastable states. Thus, in the phase space of this basic model there must be several (in general many) saddle states connected by unstable separatrices. Such chain represents the process of sequential switching of activity from one cognitive mode to the next one. This process can be finite, i.e., ending on a simple attractor or repetitive. If we choose the variables *A_j_*(*t*) as the amount of activation of the different modes, we can suppose that the saddle points are disposed on the axes of an *N*-dimensional phase space, and the separatrices connecting them are disposed on a (*N*−*n*)-dimensional manifold (*n*<*N*−1), which are the boundaries of the phase space.

We will use two types of models that satisfy the above conditions: (i) the Wilson-Cowan model for excitatory and inhibitory neural clusters [42], and (ii) generalized Lotka-Volterra equations – a basic model for the description of competition phenomena with many participants [32],[43]. Both models can be represented in a general form as:
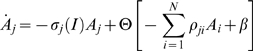
(2)


Here *A_j_*(*t*)≥0 is the activation level of the *j*-th cluster, Θ[*z*] is a nonlinear function, i.e., a sigmoid function in the case of the Wilson-Cowan model and a polynomial one for the generalized Lotka-Volterra model. The connectivity matrix ρ*_ji_* can depend on the stimulus or change as a result of learning. σ(*I*) is a parameter characterizing the dependence of the cognitive states on the incoming information *I*. The parameter β represents other types of external inputs or noise. In the general case, *A_j_* (*t*) is a vector function whose number of components depends on the complexity of the intrinsic dynamics of the individual brain blocks. The cognitive mode dynamics can be interpreted as a nonlinear interaction of such blocks that cooperate and compete with each other.

To illustrate the existence of a stable heteroclinic channel in the phase space of Equation 2, let us consider a simple network that consists of three competitive neural clusters. This network can be described by the Wilson-Cowan type model as
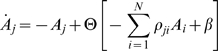
(3)where ρ_jj_<0, ρ_j≠i_≥0, β>0, *N* = 3.

The network can also be described by a Lotka-Volterra model of the form:
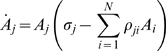
(4)where ρ_ji_≥0. In all our examples below we will suppose that the connection matrix is non symmetric, i.e., ρ*_ji_*≠ρ*_ij_*, which is a necessary condition for the existence of the SHC.

**Figure 2:**
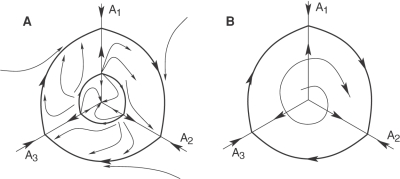
Closed stable heteroclinic sequence in the phase space of three coupled clusters. (A) Wilson-Cowan clusters. (B) Lotka-Volterra clusters.

Figure 2 illustrates how the dynamics of these two models with *N* = 3 can produce a robust sequential activity: both models have SHC in their phase-spaces. The main difference between the dynamics of the Wilson-Cowan and Lotka-Volterra models is the type of attractors. System 3 contains a stable limit cycle in a SHC and a stable fixed point (the origin of the coordinates for β = 0). In contrast, there is one attractor, i.e., a SHC, in the phase space of System 4.

**Figure 3:**
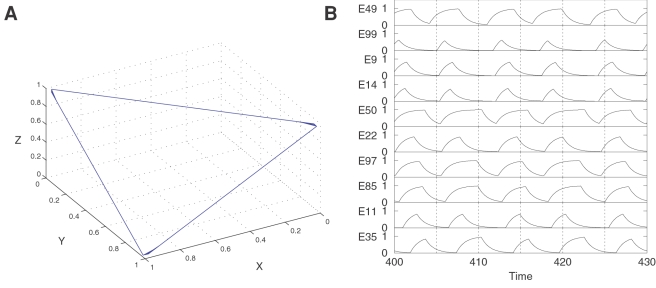
Robust transient dynamics of 200 cognitive modes modeled with Wilson-Cowan equations. (A) The activation level of three cognitive modes are shown (E14, E11, E35), (B) Time series illustrating sequential switching between modes: 10 different modes out of the total 200 interacting modes are shown.

Both models demonstrate robust transient (sequential) activity even for many interacting modes. An example of this dynamics is presented in Figure 3. This figure shows the dynamics of a two-component Wilson-Cowan network of 100 *excitatory* and 100 *inhibitory modes*. The parameters used in these simulations are the same as those reported in [44] where the connectivity was drawn from a Bernoulli random process but with the probability of connections slightly shifted with respect to the balanced excitatory-inhibitory network. The system is organized such that a subgroup of modes fall into a frozen component and the rest produce the sequential activity. The model itself is sufficiently general to be translated to other concepts and ideas as the one proposed here in the form of cognitive modes.

**Figure 4:**
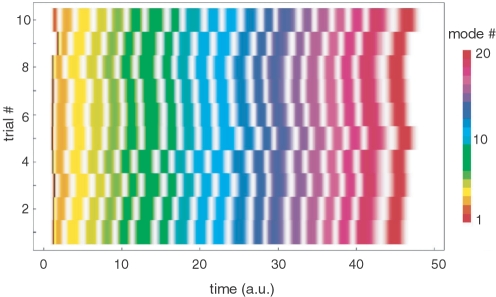
Reproducibility of a transient sequential dynamics of 20 metastable modes corresponding to SHC in Model 4. The figure shows the time series of 10 trials. Simulations of each trial were initiated at a different random initial condition. The initial conditions influence the trajectory only at the beginning due to the dissipativeness of the saddles (for details see also [10]).

Figure 4 illustrates the reproducibility of transient sequential dynamics of Model 4 with *N* = 20 modes. This simulation corresponds to the following conditions: (i) ρ*_ji_*≠ρ*_ij_* and (ii) *v_i_*>1 (see [10] for details). In this figure each mode is depicted by a different color and the level of activity is represented by the saturation of the color. The system of equations was simulated 10 times, each trial starting from a different random initial condition within the hypercube 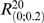. Note the high reproducibility of the sequential activation among the modes, which includes the time interval between the switchings.

Because of the complexity of System 4 with large *N*, the above conditions cannot guarantee the absence of other invariant sets in this system. However we did not find them in our computer simulations. For a rigorous demonstration of the structural stability of the SHC see Methods section.

It is important to emphasize that the SHC may consist of saddles with more than one unstable manifold. These sequences can also be feasible because, according to [40] and [45], if a dynamical system is subjected to the influence of small noise, then for any trajectory going through an initial point in a neighborhood of such saddle, the probability to escape this neighborhood following a strongly unstable direction is almost one. The strongly unstable direction corresponds to the maximal eigenvalue of the linearization at the saddle point. In other words, everything occurs in the same way as for the SHC; one must only replace the unstable separatrices in the SHC by strongly unstable manifolds of saddle points.

As we mentioned above, the variables *A_i_*(*t*)≥0 in the basic Equations 2 or 4 can be interpreted in several different ways. One of them which is related to experimental work is the following. Using functional Principal Component (PC) analysis of fMRI data (see, for example [46]) it is possible to build a cognitive “phase space” based on the main orthogonal PCs. A point in such phase space characterizes the functional cognitive state at instant *t*. The set of states in subsequent instants of time is a cognitive trajectory that represents the transient cognitive dynamics.

### Sequential Decision Making

Decisions have to be reproducible to allow for memory and learning. On the other hand, a decision making (DM) system also has to be sensitive to new information from the environment. These requirements are fundamentally contradictory, and current approaches [47]–[50] are not sufficient to explain the use of sequential activity for DM. Here, we formulate a new class of models suitable for analyzing sequential decision making (SDM) based on the SHC concept, which is a generalization of Model 4.

A key finding in Decision Theory [51] is that the behavior of an individual shifts from risk-aversion (when possible gains are predicted) to risk seeking (when possible losses are predicted). In particular, Kahneman and Tversky [52] conducted several experiments to test decision making under uncertainty. They showed that when potential profits are concerned, decision-makers are risk averse, but when potential losses are concerned, subjects become risk seeking. Other classical paradigms assume that decision makers should always be risk averse, both when a potential profit and when a possible loss are predicted.

#### SDM model

To illustrate how the SHC concept can be applied to the execution of a specific cognitive function, let us consider a simple fixed time (T*) game: a player is taking sequential actions in a changing environment so as to maximize the reward. The success of the game depends on the decision strategy. Formally, the SDM model consists of: (i) a set of environment states σ(*I*); (ii) a set of dynamical variables *A_j_*≥0 characterizing the level of activity of the cognitive modes that correspond to the execution of the decision strategy; and (iii) a scalar representing the cumulative reward that depends on the number of achieved steps in the available time T*, and on the values of the instantaneous reward at the steps along different transients, i.e., different choices. Depending on the environment conditions, the game can end at step (*k*+1), or it can continue using one or many different ways based on the different choices. It is clear that to get the maximum cumulative reward the player has to pass as many steps within the game's time T*. Thus, the strategy that will make the game successful has to be based on two conditions:

the game does not have to end in an attractor (stable fixed point) at time t<T*, and
the player has to encounter as many metastable states as possible during the time T*.

#### Strategy

It is difficult to estimate analytically which strategy is the best to solve the first problem. It can be done in a computer simulation, but we can make a prediction for the second problem. Let us assume that we have a successful game and, for the sake of simplicity, that the reward on each state is identical (as our computer simulations indicate, the results do not qualitatively change if the rewards for each step are different). Thus, the game dynamics in the phase space can be described by the system
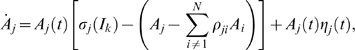
(5)

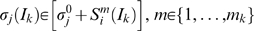
(6)where *A_j_*≥0, *m_k_* is the number of admissible values of σ*_j_* at the decision step *t_k_*, 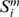 represents the stimulus determined by the environment information *I_k_* at the step *t_k_*, and η*_j_* is a multiplicative noise. We can think that the game is a continued process that is represented by a trajectory arranged in a heteroclinic channel (see Figure 1). The saddle vicinities correspond to the decision steps. Evidently, the number of such steps increases with the speed of the game that depends on the time that the system spends in the vicinity of the saddle (metastable state) as given by Equation 1: *t_k_* = 1/λ*_k_* ln (1/|η|) where |η| is the level of perturbation (average distance between the game trajectory and the saddle at decision step *t_k_*), and λ*_k_* is a maximal increment that corresponds to the unstable separatrices of this saddle. From this estimate we can make a clear prediction. If the system does not stop in the middle of the game (see Problem 1 above), to get the best reward a player has to choose the σ(*I_k_*) that correspond to the maximal λ*_k_* and to have an optimal level of the noise (not too much to avoid leaving the heteroclinic channel). Suppose that we have noise in the input *I* that controls the next step of the decision making. Since
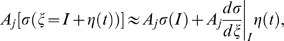
(7)such additive informational noise appears on the right side of the dynamical model as a multiplicative noise.

#### Computer modeling

The parameters of the model were selected according to a uniform distribution in the range 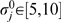. As a proof of concept, the specific order of the sequence is not important. Therefore, the sequence order is set from saddle 0 to *N* which is obtained by setting a connectivity matrix so that 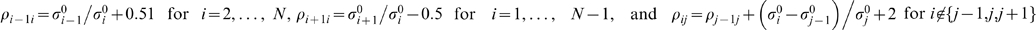. Note that there are infinite matrices that will produce the same sequence. All the rest of the parameters that form the basis of all possible perturbations or stimulations at each of the saddles or decision steps were taken from a uniform distribution 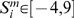. The specific selection of these parameters does not have any impact on the results that are shown throughout this paper. For the sake of simplicity, we assume that the external perturbations at each of the decision steps are uncorrelated. The dynamical systems 5 and 6 was integrated using a standard explicit variable Runge-Kutta method.

When the trajectory reaches the vicinity of a saddle point within some radius ε = 0.1, then the decision making function is applied. The rule applied in this case is the high-risk rule, which is implemented as follows. At each saddle we calculate the increments λ*_j_*_(*q*)*i*_ = σ*_j_*_(*q*)_−ρ*_j_*_(*q*)*i*_σ*_j_*_(*q*)_ with *q* = 1,…, *m_k_* such that a specific *q* is chosen to obtain a maximal λ*_j_*_(*q*)*i*_ at each saddle. In other words, we choose the maximal increment, which corresponds to the fastest motion away from the saddle *S_i_*, and therefore, the shortest time for reaching the next saddle.

**Figure 5:**
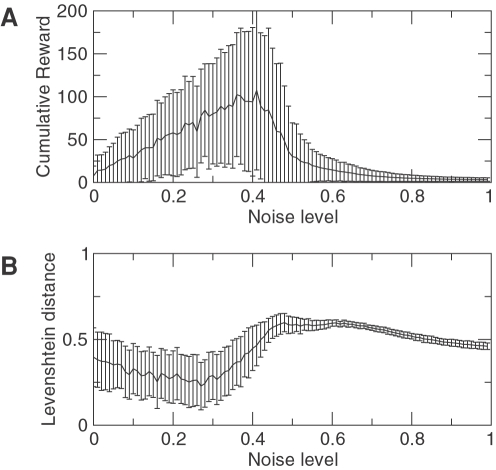
Estimation of the cumulative reward for different noise levels using multiplicative noise. (A) Cumulative reward calculated as the number of cognitive states that the system travels through until the final time of the game T* which is 100 in this case. For each level of noise, 1000 different sequences are generated (for *N* = 15 and a total of 15 choices). (B) Reproducibility index of the sequence calculated with the average Levenshtein distance across all generated sequences. The lower the distance, the more similar the sequences are for 1000 different runs. The pair distances are calculated and averaged to obtain the mean and the standard deviation which is represented by the error bars.

To evaluate the model, we analyzed the effect of the strength of uncorrelated multiplicative noise 〈η*_j_*(*t*)η*_j_*(*t*′)〉 = μδ(*t*−*t*′). The results are shown in Figure 5. As the theory predicted, the noise plays a key role in the game, and there exists an optimal level of noise. For low noise the system travels through most of the saddles in a slower manner (see Equation 1), while for increasing values of the noise the number of metastable states involved in the game are reduced. Figure 5A shows the cumulative reward for different noise levels. Two interesting cases were investigated. As we can see from the figure, the optimal cumulative reward is obtained for a particular noise level. For levels of moderate noise the system enters partially repeated sequences, because the two or more unstable directions allow the system to move to two or more different places in a random fashion. The reproducibility measure of the obtained sequences is shown in Figure 5B. We can see that the most reproducible sequences are generated for a slightly smaller level of noise than the one that corresponds to the maximum cumulative reward. To estimate the reproducibility across sequences we used the Levenshtein distance that basically finds the easiest way to transform one sequence into another [53]. This distance is appropriate to identify the repetitiveness of the sequence and it is used in multiple applications. Sometimes it happens that the sequence becomes repetitive, and in other cases it just dies. The error bars in this figure denote the standard deviation. While the Levenshtein distance displays not too large error bars, the cumulative reward does because for that level of noise is common to enter limit cycles that reach the maximum time. It is more likely to find two extremes: (i) ending quickly and (ii) reaching a limit cycle.

Concerning the formation of a habit it is important to note that the memorized sequence is subjected to the external stimulation that can change the direction at any given time. This fact is reflected in the results shown in Figure 5 where the Levenshtein distance does not go exactly to zero. The heteroclinic skeleton that forms the SHC can be broken and can even repeat itself to produce limit cycles for a given set of external stimulus. So the model does have alternatives that are induced by the set of external perturbations under the risk taking decision making rule.

This simple game illustrates a type of transient cognitive dynamics with multiple metastable states. We suggest that other types of sequential decision making could be represented by similar dynamical mechanisms.

## Discussion

We have provided in this paper a theoretical description of the dynamical mechanisms that may underlie some cognitive functions. Any theoretical model of a very complex process such as a cognitive task should emphasize those features that are most important and should downplay the inessential details. The main difficulty is to separate one from another. To build our theory we have chosen two key experimental observations: the existence of metastable cognitive states and the transitivity of reproducible cognitive processes. We have not separated the different parts of the brain that form the cognitive modes for the execution of a specific function. The main goal of such coarse grain theory is to create a general framework of transient cognitive dynamics that is based on a new type of model that includes uncertainty in a natural way. The reproducible transient dynamics based on SHC that we have discussed contains two different time scales, i.e., a slow time scale in the vicinity of the saddles and a fast time scale in the transitions between them (see Figure 1). Taking this into account, it is possible to build a dynamical model based not on ODEs but on a Poincare map (see for a review [5]), which can be computationally very efficient for modeling a complex system.

Winnerless competitive dynamics (represented by a number of saddle states whose vicinities are connected by their unstable manifolds to form a heteroclinic sequence) is a natural dynamical image for many transient cognitive activities. In particular we wish to mention transient synchronization in the brain [54], where authors have studied the dynamics of transitions between different phase-synchronized states of alpha activity in spontaneous EEG. Alpha activity has been characterized as a series of globally synchronized states (quasi-stable patterns on the scalp). We think that this dynamics can be described on the framework of the winnerless competition principle. From the theoretical point of view, a heteroclinic network between partially synchronized phase clusters has been analyzed in [55],[56]. The SHC concept allows considering transitions even between synchronized states with strongly different basic frequencies (like gamma and beta frequencies).

Cognitive functions can strongly influence each other. For example, when we model decision making we have to take into account attention, working memory and different information sources. In particular, the dynamic association of various contextual cues with actions and rewards is critical to make effective decisions [57]. A crucial question here is how to combine several reward predictions, each of which is based on different information: some reward predictions may only depend on visual cues, but others may utilize not only visual and auditory cues but also the action taken by a subject. Because the accuracy of different reward predictions varies dynamically during the course of learning, the combination of predictions is important [58]. In a more general view, the next step of the theory has to be the consideration of mutual interaction of models like Model 4 that represent the execution of different cognitive functions.

The dynamical mechanisms discussed in this paper can contribute to the interpretation of experimental data obtained from brain imaging techniques, and also to design new experiments that will help us better understand high level cognitive processes. In particular, we think that the reconstruction of the cognitive phase space based on principal component analysis of fMRI data will allow finding the values of the dynamical model parameters for specific cognitive functions. To establish a direct relation between model variables and fMRI data will be extremely useful to implement novel protocols of assisted neurofeedback [59]–[62], which can open a wide variety of new medical and brain-machine applications.

## Methods

### Stable Heteroclinic Sequence

We consider a system of ordinary differential equations
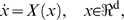
(M1)where the vector field *X* is *C* ^2^-smooth. We assume that the system M1 has *N* equilibria *Q_1_*, *Q_2_*, …, *Q_N_*, such that each *Q_i_* is a hyperbolic point of saddle type with one dimensional unstable manifold 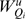 that consists of *Q_i_* and two “separatrices”, the connected components of 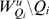 which we denote by 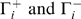. We assume also that 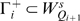, the stable manifold of *Q_i_*_+1_.

#### Definition

The set 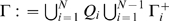 is called the heteroclinic sequence.

We denote by 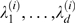 the eigenvalues of the matrix 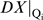. By the assumption above one of them is positive and the others have negative real parts. Without loss of generality one can assume that they are ordered in such a way that
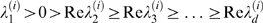



We will use below the saddle value (see Equation 1)
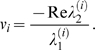



For readers who are interested in understanding the details of these results we recommend, as a first step, to read references [63],[64].

#### Definition M1

*The heteroniclic sequence* Γ *is called the stable heteroclinic sequence (SHS) if*

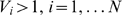
(M2)


It was shown in [10],[32] that the conditions M2 imply stability of Γ in the sense that every trajectory started at a point in a vicinity of *Q*_1_ remains in a neighborhood of Γ until it comes to a neighborhood of *Q_N_*. In fact, the motion along this trajectory can be treated as a sequence of switchings between the equilibria *Q_i_*_ = 1, 2,…,*N*_

Of course, the condition 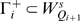 indicates the fact that the system M1 is not structurally stable and can only occur either for exceptional values of parameters or for systems of a special form. As an example of such a system one may consider the generalized Lotka-Volterra Model 4 (see [10],[32]).

### Stable Heteroclinic Channel

We consider now another system, say,
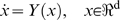
(M3)that also has *N* equilibria of saddle type *Q_1_*, *Q_2_*, …, *Q_N_* with one dimensional unstable manifold 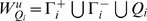, and with *v_i_*>1, *i* = 1,…,*N*. Denote by *U_i_* a small open ball of radius ε centered at *Q_i_* (one may consider, of course, any small neighborhood of *Q_i_*) that does not contain invariant sets but *Q_i_*. The stable manifold 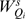 divides *U_i_* into two parts: 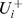 containing a piece of 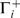, and another one 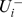. Assume that 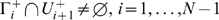, and denote by 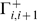 the connected component of 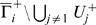 containing *Q_i_* and that 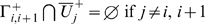. Denote by 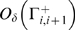 the δ-neighborhood of 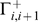 in ℜ^d^.

#### Definition M2

*Let* 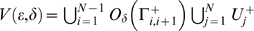. *We say that the System M3 has a stable heteroclinic channel in V*(ε,δ) *if there exits a set* 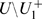 *of initial points such that for every x*_0_ ⊂ *U there exits T*>0 *for which the solution x*(*t*,*x*_0_), 0≤*t*≤*T*, *of M3 satisfies the following conditions*:

- *x*(0, *x*_0_) = *x*_0_
- *for each* 0≤*t*≤*T*, *x*(*t*,*x*_0_) ∈ *V*(ε,δ)
- *for each* 1≤*i*≤*N there exists t_i_*<*T such that* 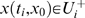

Thus, if ε and δ are small enough, then the motion on the trajectory corresponding to *x*(*t*,*x*_0_) can be treated as a sequence of switchings along the pieces 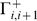 of unstable separatrices between the saddles *Q_i_*, *i* = 1,…,*N*.

It follows that the property to possess a SHC is structurally stable: if a System M3 has a SHC then a *C* ^1^- close to System M3 also has it.

We prove this fact here under additional conditions. Denote by 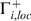 the intersection 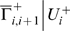. It is a segment for which one end point is *Q_i_* while the other one, say *P_i_*, belongs to the boundary 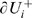. Let 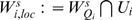, the piece of the stable manifold of *Q_i_* and 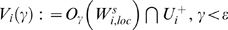, where *O*_γ_(*B*) is the γ-neighborhood of a set *B* in ℜ^d^. The boundary ∂*V_i_*(γ) consists of 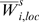, a (*d*-1)-dimensional ball, *B_i_*, “parallel to” 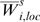 and a “cylinder” homeomorphic to *S ^d^* ^−2^×*I*, where *S ^d^* ^−2^ is the (*d*-2)-dimensional sphere and *I* is the interval [0,1]. We denote by *C_i_* (γ) this cylinder. The proof of the following lemma is rather standard and can be performed by using a local technique in a neighborhood of a saddle equilibrium (see [63]–[65]).

#### Lemma M1

*There is* 0<ε_0_<1 *such that for any* ε<ε_0_ *and any* 1≤*i*≤*N there exist* ε*_i_*<ε_0_ *and* 1<μ*_i_*<*v_i_ for which the following statement holds*: *if* 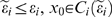 *then*

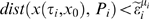
(M4)
*where* “*dist*” *is the distance in* ℜ^d^, τ*_i_*>0 *is the time and x*(τ*_i_*, *x*_0_) *is the point of exit of the solution of M3*, *going through x*_0_, *from* 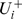.

*A segment* 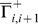 *has two end points*: *one of which is P_i_ and the other one*, *say* 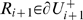. *Fix* ε<ε_0_.

#### Lemma M2

*There exists members K_i_*>1 *and* γ*_i_*>0 *such that if* 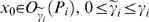, *then*:

- *there is* 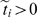 *such that* 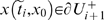

- *the following inequality holds*

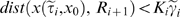
(M5)
- *every point x*(*t*, *x*_0_), 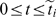 *belongs to the* 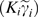-*neighborhood of* 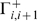.

The lemma is a direct corollary of the theorem of continuous dependence of a solution of ODE on initial conditions on a finite interval of time.

Now, fix the numbers μ*_i_*, ε*_i_* satisfying Lemma M1. Then we impose a collection of assumptions that will guarantee the existence of the SHC.

#### Assumption M_N_

*The point* 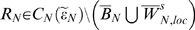.

The lemma M2 implies that there exits 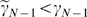 such that 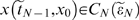 for every 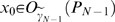. Fix a number 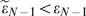 such that
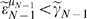
(M6)


#### Assumption M_N−1_

*The point* 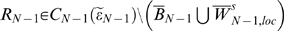.

Again, there exits 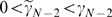 such that 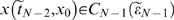 for every 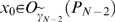. Fix a number 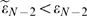 such that
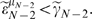
(M7)


Continuing we come to

#### Assumption M_i_

(*i* = 1,…,*N*−2) *The point* 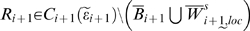.

We choose 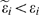 such that
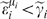
(M8)where 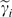 is fixed in such a way that 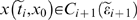 provided that 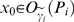.

The following theorem is a direct corollary of Lemmas 1 and 2, the assumptions *M*_N_−*M*_2_ and the choice of numbers 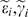:

#### Theorem M2

Under *the assumptions above*, *the System M3 has a SHC in V*(ε, δ) *where* 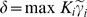 *and the set of initial points (see Definition M2)* 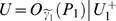.

#### Corollary

*There exists* σ>0 *such that every system*

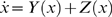

*where* 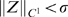 *also has a SHC in V*(ε, δ), *maybe with a smaller open set U of initial points*.

The proof of Corollary is based:

- on the fact that the local stable and unstable manifolds of a saddle point for an original and a perturbed system are *C* ^1^-close to each other;
- on the theorem of smooth dependence of a solution of ODE on parameters and
- on the open nature of all assumptions of Theorem M2.

The conditions 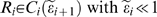 look rather restrictive, in general. Nevertheless, for an open set of perturbations of a system possessing a SHS, they certainly occur.

#### Theorem M3

*If a System M1 has a SHS then there is an open set U in the Banach space of vector fields with the C* ^1^-*norm such that the system*

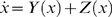

*has a SHC*, *for every Z*∈*U*.

#### Proof

The proof can be made by a rather standard construction. Since 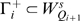 for the system (M1) then in some local coordinates around a point 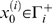 the System M1 can be written as
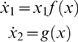
(M9)where *x*_1_∈*P*, *x*_2_∈*P* *^d^* ^−1^, *x* = (*x*_1_,*x*_2_), and the inequality *x*_1_>0 determines the side of 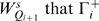 belongs to. Denote by 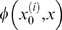 the “cup-function”: a C^1^-smooth function ℜ^d^→ℜ^+^ such that 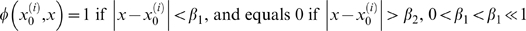. Now the system
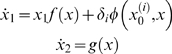
(M10)will have a piece of the separatrix 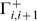 satisfying the assumption M*_i_* if 0<δ*_i_*<<1. We perturb the System M1 in such a way for every *i* = 1, …,*N*−1 and obtain a System M3 having SHC provided that all δ*_i_*>0 and sufficiently small.
